# Expression of Genes Related to Meat Productivity, Metabolic and Morphological Significance of Broiler Chickens with the Use of Nutritional Phytochemicals

**DOI:** 10.3390/ani14202958

**Published:** 2024-10-14

**Authors:** Marina I. Selionova, Vladimir I. Trukhachev, Artem Yu. Zagarin, Egor I. Kulikov, Dmitry M. Dmitrenko, Vera N. Martynova, Arina K. Kravchenko, Vladimir G. Vertiprakhov

**Affiliations:** 1 Department of Animal Breeding, Genetics and Biotechnology, Institute of Animal Science and Biology, Russian State Agrarian University—Moscow Timiryazev Agricultural Academy, 49 Timiryazevskaya Str., Moscow 127434, Russia; selionova@rgau-msha.ru; 2 Department of Animal Nutrition, Institute of Animal Science and Biology, Russian State Agrarian University—Moscow Timiryazev Agricultural Academy, 49 Timiryazevskaya Str., Moscow 127434, Russia; rector@rgau-msha.ru; 3 Laboratory of Applied Genetics, Federal State Budget Scientific Institution Federal Scientific Center “All-Russian Research and Technological Poultry Institute”, 10 Ptitsegradskaya St., Sergiev Posad, Moscow 141311, Russia; kulikovegor33@yandex.ru (E.I.K.); dmitrenko.dmitry1999@yandex.ru (D.M.D.); veramartnik@yandex.ru (V.N.M.); arinkakravchenko@yandex.ru (A.K.K.); 4 Department of Physiology, Ethology and Biochemistry of Animals, Institute of Animal Science and Biology, Russian State Agrarian University—Moscow Timiryazev Agricultural Academy, 49 Timiryazevskaya Str., Moscow 127434, Russia; vertiprahov@rgau-msha.ru

**Keywords:** blood biochemical parameters, broiler chickens, *FASN*, gene expression, *MSTN*, *MYOG*, nutrigenomics, phytobiotics

## Abstract

**Simple Summary:**

Transcriptomic studies allow us to reveal at the molecular level the nature of the influence of various paratypical factors on physiological processes underlying the formation of animal productivity and health. The uncontrolled use of feed antibiotics is one of the key reasons for the development of antibiotic resistance in pathogens, necessitating the testing of alternative nutraceuticals in animal nutrition, including phytobiotics, the effectiveness and biological properties of which can be identified with the help of modern molecular genetic biotechnologies in addition to traditional methods of phenotype assessment. The nature of changes in gene expression related to the formation of skeletal muscles and lipogenesis was studied when using biologically active substances extracted from plants in the nutrition of broiler chickens.

**Abstract:**

The study aimed to analyze gene expression linked to skeletal muscle growth and lipid metabolism in broiler chickens fed with plant extracts. Five groups of chickens were formed: four experimental groups and one control group. The diets of the experimental groups were supplemented with different plant extracts: chicory, St. John’s wort, maral root, and creeping thyme, whereas the control group received feed without phytobiotic compounds. Weekly weighings were conducted (*n* = 36). The chickens were slaughtered at day 26 for tissue sampling of four birds from each group. Gene expression (*MYOG*, *MSTN*, *FASN*) related to muscle growth and fatty acid synthesis was analyzed using the β-actin *ACTB* gene as a reference. Blood samples were taken at day 35 for biochemical analysis and anatomical dissection was performed. The study revealed that using plant extracts from chicory, thyme, and maral root increased *MYOG* gene activity by 4.21, 7.45, and 8.93 times, respectively. *T. serpyllum* extract boosted the *MSTN* gene by 10.93 times, impacting muscle growth regulation. *FASN* gene expression for fatty acid synthesis increased significantly by 18.22–184.12 times with plant extracts. The best results regarding meat productivity of chickens were obtained when using *R. carthamoides* extract. The results of the study will serve as a basis for further development of a phytocomposition designed to increase the meat productivity of broiler chickens in the production of environmentally safe poultry products.

## 1. Introduction

The use of feed antibiotics is the most common way to stimulate growth and increase productivity of food-producing animals. However, the irrational use of antibacterial agents in the feed industry is a key reason for the retention of residual amounts of antibiotics in animal products and the development of antibiotic resistance in pathogenic microorganisms [1]. The problem of antibiotic resistance is one of the major issues in the modern world, as the emergence of resistance mechanisms in bacteria to antibiotic compounds leads to a decrease in the effectiveness of treating infectious diseases in both animals and humans as consumers of animal products. According to preliminary forecasts, the increasing microbial resistance to antibiotics will result in economic costs of 100 trillion dollars by 2050 in the absence of measures to reduce it [2,3].

The above stated stipulates the need for search, testing, and implementation of nutraceuticals in animal husbandry as alternatives to feed antibiotics [4]. They include phytobiotics, non-nutritive natural biologically active components of plant origin, positively affecting the productivity and health of animals and having a number of advantages, such as low level of retention, lack of pathogen resistance development to their active components, and minimal side effects [5]. The range of phytobiotics primarily includes plant extracts, essential oils, aromatic herbs, and algae derivatives [6]. The composition of the main active substances in phytobiotics varies depending on factors such as geographical origin, season of raw material collection, combination of different plants, and their parts in one product [7].

The widespread use of phytobiotics in feeding farm animals is driven by a complex of their biologically active properties, including antibacterial, fungicidal, antiviral, immunostimulating, antioxidant properties, stimulation of digestive enzymes, and improvement of feed nutrient digestibility [8]. Furthermore, their ecological safety for animal husbandry and human consumption is noted; therefore, these products are considered as natural growth promoters (NGPs) [9]. These properties of phytobiotics were repeatedly observed in scientific experiments conducted in key sectors that support industrial meat production: broiler farming [10,11,12] and pig farming [13,14,15].

The application of genetic research methods allows for a more precise evaluation at the molecular level of the impact of paratypical factors on processes that determine the formation of animal phenotypes, primarily productivity and their resistance to unfavorable factors. By studying the transcriptional activity of individual genes or the entire transcriptome as a whole, it is possible to identify specific biologically active properties of nutraceuticals and feed ingredients that may be hidden during phenotype assessment. For example, in several scientific studies, the expression of genes in agricultural poultry associated with traits such as inflammation initiation, apoptosis, antimicrobial and antiviral activity [16], myogenesis in the embryonic development period [17], resistance to toxic and medicinal substances [18], antioxidant protection, and adaptation to heat stress was explored [19,20].

However, the influence of the level of transcriptional activity of target genes, determined by the impact of nutrients and bioactive substances in the diet, on the formation of economically valuable and biological traits was not sufficiently studied. Moreover, for poultry farming, genes associated with growth, meat productivity, and resistance to adverse factors are of particular interest, which determines the relevance of this study.

In the present study, it is established for the first time that the intensification of growth, metabolism, and increased meat productivity of broiler chickens of the “Smena-9” cross when fed with *Rhaponticum carthamoides* L. extract standardized for the content of the phytoecdysteroid ecdysterone is associated with the activation of mRNA expression of the *MYOG* and *FASN* genes, associated, respectively, with the development and differentiation of skeletal muscle and fatty acid synthesis. The increase in transcriptional activity of the *MSTN* gene, which restricts muscle growth and development, suggests the feasibility of further studies in search of a new phytocomposition for the combined use of *Thymus serpyllum* L. in order to mitigate the increase in *MSTN* gene expression.

The aim of the present study was to investigate the expression of genes related to skeletal muscle growth and lipid metabolism, the nature of growth, metabolic, and morphological characteristics of broiler chickens (*Gallus gallus* L.) when fed with biologically active components extracted from plant raw materials with different functional properties, in order to develop an effective phytobiotic composition.

## 2. Materials and Methods

The study was reviewed and approved by the Bioethics Committee of the Russian State Agrarian University—Moscow Timiryazev Agricultural Academy (protocol No. 16 dated 30 January 2024).

### 2.1. Experimental Design, Characteristics of Objects and Conditions of Research

The experiment was conducted from March to April 2024 in the vivarium of the Russian State Agrarian University—Moscow Timiryazev Agricultural Academy on broiler chickens (*Gallus gallus* L.) of the “Smena-9” cross for 35 days in accordance with the Recommendation of the Board of the Eurasian Economic Commission dated 14 November 2023, No. 33 “On the Guidelines for Working with Laboratory (Experimental) Animals in Preclinical (Non-clinical) Studies”.

After the completion of the incubation at the Center for Genetics and Selection “Zagorskoye EPH” (Sergiyev Posad, Russia) using the method of balanced group analogs considering live weight and overall development, 5 groups of 36 birds each were formed (Table 1). In the first control group, broiler chickens were fed compound feeds without the addition of phytoingredients (CON); in the second experimental group, common chicory extract (*Cichorium intybus* L.) was introduced into the feed at a rate of 450.0 g/ton in the “Starter” and “Grower” feeds and 560.0 g/ton in the “Finisher” compound feed, equivalent to 405.0 and 504.0 g of inulin per ton of “Starter”/”Grower” feeds, “Finisher”, respectively (CCE); in the third group—St. John’s wort (*Hypericum perforatum* L.) extract at a rate of 350.0 and 430.0 g/ton, equivalent to 22.6 and 31.4 g/ton of flavonoids, respectively (SJWE); in the fourth group—maral root (*Rhaponticum carthamoides* L.) extract at a rate of 170.0 and 210.0 g/ton, equivalent to 0.9 and 1.1 g/ton of the phytoesteroide ecdisten, respectively (MRE); and in the fifth experimental group—creeping thyme (*Thymus serpyllum* L.) extract at a rate of 200.0 and 250.0 g/ton, equivalent to 36.8 and 46.0 g/ton of flavonoids and 7.20 and 9.00 g/ton of tannins in the “Starter”/”Grower”, “Finisher” feeds, respectively (TCE). The rate of introduction of extracts into compound feeds was selected for the first time. However, the results of earlier studies were taken into account when developing the experimental methodology [21,22,23,24]. In addition, the economic criterion was the basis for the selection of the extract introduction rate: the cost of extracts introduced into the feed in all groups was equivalent to USD 5.52 (500 Russian rubles) per 1 tonne of feed at the time of methodology development.

The birds were kept in wired floor cages following the temperature–humidity regime, stocking density, and lighting schedule suggested by the cross developers [25]. The room temperature was maintained at 31–33 °C during the first week of the chickens’ life, 24–30 °C during the second and third weeks, and 20–23 °C during the fourth and fifth weeks of the experiment. Relative humidity ranged from 40–60% in the first week to 60–70% for the rest of the experiment. The lighting regime was maintained using a timer according to the following scheme: on the day of the end of incubation and caging of the young—constant light, days 1–7—23 h of light and 1 h of darkness, days 8–34—4 cycles of 5 h of light and 1 h of darkness, and day 35–23 h of light and 1 h of darkness. The cages were three-tiered structures, the length area of a separate section being 65 cm × 70 cm. The chickens were placed in sections in such a way that the caging density by the end of the experiment did not exceed 476 cm^2^ per head (no more than 21 heads per m^2^). Chickens of each individual group were evenly distributed in all tiers. In the first week, paper was used as bedding, and then the birds were placed on a wire floor. The feeding and drinking regime was ad libitum. Feeding was carried out using hopper-type and trough feeders, while drinking was provided through vacuum and nipple drinkers depending on the age of the birds.

### 2.2. Broiler Chicken Nutrition and Phytobiotic Feed Additives

Throughout the experiment, complete phase compound feeds were used: from the moment of hatching to day 10—“Starter”, from day 11 to 22—“Grower” in pellet form, and from day 23 to 35—“Finisher” in crumble form. The transition to crumbles in the final phase of rearing was due to economic feasibility, which is allowed by the guidelines for working with poultry of this cross [25]. The producer of the compound feed was JSC “Melkombinat” (Tver, Russia). The conditions of compound feed production, including its pelleting, corresponded to the parameters accepted at the factory. Guar powder was used as a thickener during pelleting. The nutritional value and chemical composition of the compound feeds corresponded to the recommendations for rearing the cross [25] (Table 2).

The aqueous extracts were dry, fine-dispersed powders of white (*C. intubus* L.), light brown (*H. perforatum* L., *T. serpyllum* L.), and dark brown (*R. carthamoides* L.) colors. Extracts of common chicory and maral root were prepared from root raw materials, and extracts of perforate St. John’s wort and creeping thyme were obtained from plant stems. The choice of the above-mentioned plants as raw materials for extracts was due to various biologically active components and their properties. Thus, inulin was considered as a prebiotic designed to stabilize the intestinal microbiota, ecdisten, as a phytosteroid that promotes the growth of muscle mass of chickens, flavonoids, and tannins as antimicrobial, antioxidant, and immunomodulatory compounds. Further work will be aimed at developing a phytocombination that will include the described extracts. The studied extracts were introduced into the feed composition using a step-by-step blending method: the amount of each extract was thoroughly mixed in 100 g of compound feed, then the obtained blend was mixed in 1 kg of compound feed (100 g of compound feed with extract +900 g of ordinary compound feed), then in 10 kg (1 kg of compound feed with extract +9 kg of ordinary compound feed), then this mixture was stirred in 50 kg of feed (10 kg of compound feed with extract +40 kg of ordinary compound feed), which allowed us to achieve a uniform distribution of the extracts in compound feed.

### 2.3. Sample Collection

For the analysis of gene relative expression at 26 days of age, tissue samples of pectoral muscles were collected from four broiler chickens from each group after euthanasia under sterile conditions with prior treatment of instruments and working surfaces using RNAseClean Soft (Biomedical Innovations, Rostov-on-Don, Russia) for decontamination from RNase. The samples were stabilized using IntactRNA fixative (“Evrogen”, Moscow, Russia) and stored at −20 °C prior to the laboratory analysis.

At 35 days of age, after a 10 h fasting period, six animals (three roosters and three hens) with an average live weight per group were selected. Their digestive system was emptied. Blood was drawn from all six birds in each group, then they were autopsied and anatomically dissected. Blood was collected from the ulnar cutaneous (*cutanea ulnaris*) vein on the inner side of the wing above the elbow joint into tubes with a clot activator (2–3 mL) [26].

### 2.4. Growth and Anatomical Features of Broiler Chickens

To assess the zootechnical efficiency of broiler chicken breeding, individual bird weighing (*n* = 36) was conducted weekly (0, 7, 14, 21, 28, and 35 days) using electronic laboratory scales Mercury 122ACFJR-600.01 (Mertech, Shchyolkovo, Russia), with an accuracy of 0.01 g, and electronic scales M-ER 223 AC-15.2 Mary LCD (Mertech, Shchyolkovo, Russia), with an accuracy of 2 g depending on the bird’s age. The live weight (g) was recorded in individual weighing forms.

According to the methodology proposed by the All-Russian Scientific Research and Technological Institute of Poultry [27], slaughter and anatomical dissection of the birds were performed. The conditions for euthanizing the birds complied with the Recommendations of the Board of the Eurasian Economic Commission dated 14 November 2023, No. 33 “On the Guidelines for Working with Laboratory (Experimental) Animals in Preclinical (Non-clinical) Studies”.

The anatomical dissection of carcasses was carried out in a specially equipped room using dissection tools, and the analysis results were recorded in protocols. Weighing of internal organs, muscles, and bones was performed using the laboratory scale Mercury 122ACFJR-600.01 (Mertech, Shchyolkovo, Russia), with an accuracy of 0.01 g.

### 2.5. Relative Gene Expression Analysis

#### 2.5.1. Isolation of RNA and Synthesis of cDNA

Molecular genetic studies were conducted at the laboratory of applied genetics of the Federal State Budget Scientific Institution Federal Research Center “All-Russian Scientific Research and Technological Institute of Poultry”. After tissue homogenization with the Bioprep-24 instrument (Allsheng, Hangzhou, China), total RNA was isolated from tissue samples with the RNA Solo kit (Eurogen, Moscow, Russia). Reverse transcription was carried out using the Magnus kit (Eurogen, Moscow, Russia) and GeneExplorer GE-96G thermal cycler (Bioer, Hangzhou, China). The concentration analysis of the isolated RNA and synthesized cDNA was performed using the Fluo-200 fluorimeter (Allsheng, Hangzhou, China) and the QuDu ssDNA kit (Thermo Fisher Scientific, Waltham, MA, USA).

#### 2.5.2. Quantitative Polymerase Chain Reaction, or Real-Time Polymerase Chain Reaction (qPCR)

The qPCR was performed using the QuantStudio 5 instrument (Thermo Fisher Scientific, Waltham, MA, USA) and the 5X qPCRmix-HS SYBR kit (Eurogen, Moscow, Russia). The housekeeping gene encoding the β-actin protein *ACTB* served as the reference gene. Amplification mode: 3 min at 95 °C (preliminary denaturation); 30 s at 95 °C, 30 s at 60 °C, and 30 s at 72 °C (40 cycles). cDNA samples were placed in triplicates. All laboratory manipulations were carried out in accordance with the protocols provided by the manufacturers of the kits for molecular genetic research. The assessment of the relative expression level was performed using the 2^−ΔΔCT^ method [28]. Specific primer pairs for gene expression analysis are presented in Table 3. The primers were selected based on a review of the world’s scientific literature and validated using Ensembl Genomic Browser.

### 2.6. Serum Biochemical Analysis

Biochemical analysis of the centrifuged serum from the Mikro 220 instrument (Hettich North America, Beverly, MA, USA) was carried out on a semi-automatic biochemical analyzer HTI BioChem SA and an automatic biochemical analyzer BioChem FC-120 (High Technology Inc., North Attleborough, MA, USA), using HTI Technology reagents. The concentration of total protein, uric acid, triglycerides, calcium, phosphorus, trypsin activity, AST, ALT, lipase, amylase, and alkaline phosphatase was investigated.

### 2.7. Statistical Analysis

Statistical data processing was carried out using the IBM SPSS Statistics 23 software (version 2023) with the application of one-way analysis of variance (ANOVA) using Tukey’s test regarding growth parameters, anatomical dissection, blood biochemical analysis, and relative gene expression of the experimental groups. Student’s *t*-test for ΔCt values was used to compare the gene expression of the experimental groups relative to the control. A difference was considered significant at *p* ≤ 0.05. The tables present the means (M) and standard errors of the means (±SEM).

## 3. Results

### 3.1. Individual Consumption of Phytochemicals by Chickens

Accounting for feed costs in the groups throughout all growth phases allowed to determine the average level of consumption of bioactive substances from plant extracts, which amounted to 1628 mg of inulin (CCE group), 97 mg of flavonoids (SJWE group), 4 mg of ecdysterone (MRE group), and 146 mg of flavonoids and 29 mg of tannins (TCE group).

### 3.2. Growth Performance of Broiler Chickens

The analysis of the dynamics of broiler chicken body weight revealed that all the phytobiotics used had growth-promoting effects to some extent, however, the degree of this effect was different (Table 4).

A more pronounced positive effect at all ages was observed when using ecdysterone, which is a part of the extract of *Rhaponticum carthamoides*. The greatest effect was noted in the initial period of ontogenesis (the starting and first half of the growth phase). Thus, the body weight of broiler chickens in the experimental group MRE at 7 and 14 days of age was higher than in the CON group by 13.9% and 9.4% (*p* ≤ 0.05), respectively. When using other phytobiotics (inulin, flavonoids, tannins in the form of extracts from common chicory, perforate St. John’s wort, and creeping thyme), the impact on broiler chicken body weight dynamics was less noticeable, with superiority over the control group ranging from 3.4 to 6.9%, but not statistically significant, although in some cases the difference was close to the statistically significant level.

### 3.3. Expression of Genes Associated with Meat Productivity

#### 3.3.1. Expression of Genes Associated with Myocyte Growth and Development

**Figure 1:**
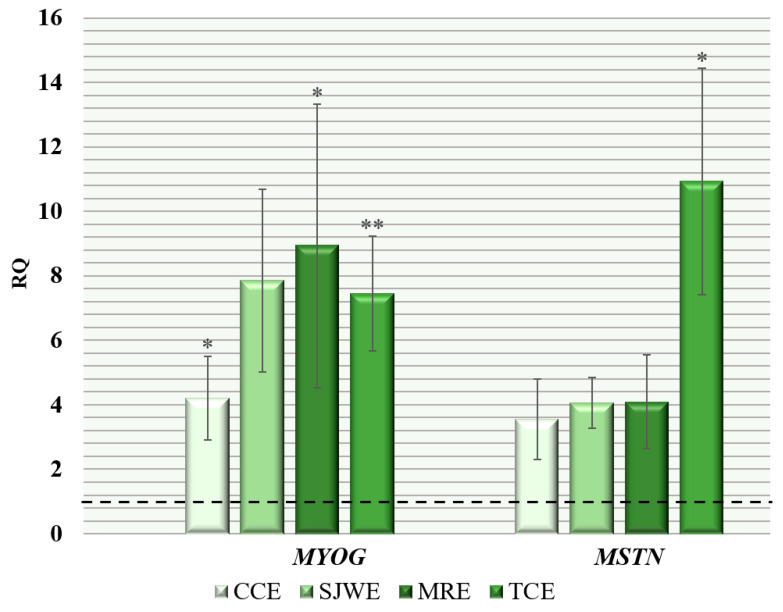
Relative quantification (RQ) of expression level of genes related to the growth and development of skeletal muscle tissues in breast muscles of broiler chickens (*Gallus gallus* L.) of the “Smena 9” cross when using phytobiotics in the diet: (RQ—fold change in expression level compared to the first group, where the parameter was taken as 1; *, **—the difference in ΔCt values is statistically significant compared to the control group at *p* ≤ 0.05, *p* ≤ 0.01 according to the t-criterion; the horizontal dashed line indicates the level of gene expression in the control group; results are presented as the mean with the standard error of the mean (M ± SEM) for mRNA expression).

The results of the gene expression study in muscle tissue of *MYOG* and *MSTN* allow us to obtain data, to some extent, explaining the differences in the varying levels of impact of the main biologically active components of the phytobiotics used on the body weight of broiler chickens (Figure 1).

In the present study, the greatest increase in comparison with the CON transcriptional activity of the *MYOG* gene was observed in the MRE group: 8.93 times (*p* ≤ 0.05). However, it should be noted that this parameter was characterized by high variability within the group, Cv = 98.4%, indicating significant differences in the individual response of chickens to ecdysterone.

In the groups of chickens receiving extracts of creeping thyme (TCE) and common chicory (CCE), the increase in *MYOG* transcriptional activity was 7.45 and 4.21 times, respectively, with less variability: Cv = 47.9% and 61.6%, respectively.

Analysis of *MSTN* expression data showed a significant increase, by 10.93 times in the group of chickens receiving creeping thyme extract (TCE), while in other experimental groups, this increase ranged from 3.56 to 4.10 times.

#### 3.3.2. Gene Expression Associated with Lipogenesis

**Figure 2:**
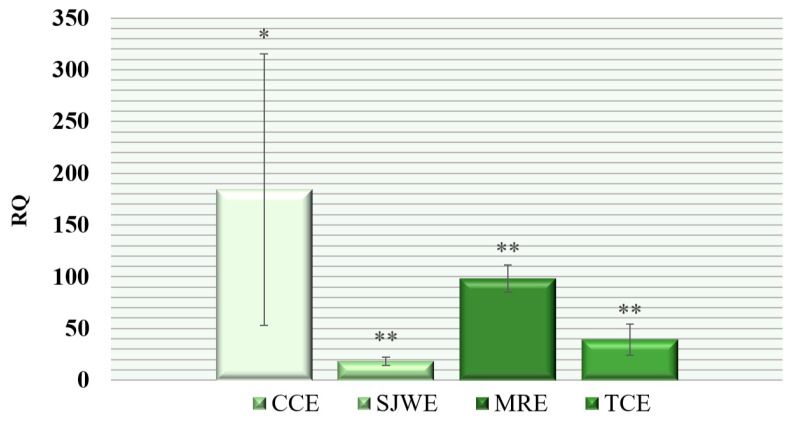
Relative quantification (RQ) of expression level of the *FASN* gene in the breast muscle tissues of broiler chickens (*Gallus gallus* L.) of the “Smena 9” cross when using phytobiotics in the diet: (RQ—fold change in expression level compared to the first group, where the parameter was taken as 1; *, **—the difference in ΔCt values is statistically significant compared to the control group at *p* ≤ 0.01, *p* ≤ 0.001 according to the t-criterion; results are presented as the mean with the standard error of the mean (M ± SEM) for mRNA expression).

The comparison of the activity of the *FASN* gene, regulating the biosynthesis of long-chain saturated and unsaturated fatty acids de novo [31], in the experimental groups revealed that the greatest increase in expression was observed in the muscle tissue of broiler chickens receiving chicory common inulin (CCE) and maral root (MRE) in 184.12 times (*p* ≤ 0.01) and 98.22 times (*p* ≤ 0.001), respectively. In the first case, a fairly high variability of the trait was noted (Cv = 142.2%), while in the second case, it was significantly lower (Cv = 26.8%). The lowest gene expression was observed in the group receiving St. John’s wort extract (SJWE)—18.22 times (*p* ≤ 0.001; Cv = 45.4%) (Figure 2).

### 3.4. Anatomical Features of Broiler Chickens

Of particular interest is comparing the expression of the genes examined with the live weight of broiler chickens at the end of the growing period and the results of their anatomical dissection (Table 5).

A tendency of superiority (by 13.2%) of birds in the MRE group receiving ecdysteroids in the form of *Rhaponticum carthamoides* plant extract in skeletal muscle mass compared to the control group was established. Greater fat deposition was observed in the groups of chickens receiving *Hypericum perforatum* flavonoids (SJWE) (by 22.1%, *p* ≤ 0.05), *Rhaponticum carthamoides* ecdysteroids (MRE) (by 18.3%, *p* = 0.074), and a complex of flavonoids and tannins from *Thymus serpyllum* (TCE) (by 19.4%, *p* = 0.052). The obtained results seem quite logical when correlated with the activity of the *MYOG*, *MSTN*, and *FASN* genes.

### 3.5. Biochemical Blood Parameters of Broiler Chickens

The assumptions about the nature of the influence of the studied genes on the performance indicators of broilers are partially confirmed by the results of the analysis of blood biochemical parameters (Table 6).

In the MRE group, where the highest live weight of broilers and the mass of skeletal muscles in their carcasses were observed, as well as high expression of *MYOG* and *FASN* genes, the lowest levels of uric acid (by 81.5%, *p* ≤ 0.05), triglycerides (by 30%), and alkaline phosphatase activity (by 73%) were noted. At the same time, the highest activity of amylase (by 49.4%) was observed, indicating a high level of protein, lipid, mineral, and carbohydrate metabolism.

## 4. Discussion

The more pronounced effect of *Rhaponticum carthamoides* extract on broilers’ live weight gain can be explained by the functional properties of the key bioactive compound, ecdysterone, belonging to phytosteroids. Their main mechanism of effect is the activation of protein biosynthesis in various tissues of the body [32]. Growth-stimulating properties of *R. carthamoides* were established in pigs, with the superiority in live weight gain at different inclusion doses of the phytobiotic ranging from 24 to 33% [33].

The positive effect of common chicory extract on live weight is apparently due to the function of inulin, which is a natural prebiotic capable of promoting significant growth of *Lactobacillus* and *Bifidobacterium* in the bird’s intestine [34]. The effect of creeping thyme and St. John’s wort extracts is probably due to the presence of flavonoids and tannins, for which antimicrobial, anti-inflammatory, hepatoprotective activity, antidiabetic, antihypertensive and immunomodulatory effects, and metabolic normalization were established [35,36].

It is known that *MYOG* is a gene encoding the protein myogenin, belonging to the family of myogenic regulatory factors (MRF), associated with proliferation and differentiation of myoblasts, and ensuring the growth and development of skeletal muscles in animals, including in the postnatal period of ontogenesis [18,37,38]. The main function of the myostatin gene (*MSTN*) is to limit the growth of muscle cells, called myocytes. Mutations in this gene lead to hyperplasia of muscle fibers in the embryonic period and their hypertrophy in the post-embryonic periods of ontogenesis [39]. Therefore, myostatin is widely used as the most promising gene in marker-associated selection and biotechnology of cattle [40], sheep [41], poultry [42], aquatic organisms reared in aquaculture [43], and other animals.

Enhancing the transcriptional activity of *MYOG* correlates with the growth performance and carcass yield results of poultry. The highest level of *MYOG* expression was observed when phytoecdysteroid ecdisten was used in the diet of chickens (MRE), which were distinguished by the highest live weight throughout the entire rearing period. The increase in *MYOG* expression when using phytosteroids in the diet was established in the study by Naji et al., which indicated an over twofold increase in the expression of this gene under the influence of the polyhydroxy phytosterol castasterone [44].

It was found that in the CCE, SJWE, and MRE groups, there is a common pattern: increased myogenin activity is accompanied by relatively low myostatin expression, while in the TCE group, simultaneous high activity of myogenin and myostatin is noted, with the latter significantly prevailing over the former. The simultaneous increase in myogenin and myostatin expression in broilers when using a prebiotic based on Chinese yam polysaccharides was identified in the study by Deng et al. [45].

It should be noted that the impact of various dietary factors on the expression of the *FASN* gene in broiler chickens was studied in several works [30,46,47], but such a significant change in transcriptional activity, as found in our study, was not observed before. This fact, in our opinion, requires further studies to elucidate the role of *FASN* when using biologically active substances of different origin in the formation of economic and biological traits.

Apparently, the increased synthesis of muscle fibers in the MRE group is due to high *MYOG* expression, while the lipocytes in the SJWE, MRE, and TCE groups exhibit high *FASN* expression. It also should be mentioned that the greater muscle mass development in the broilers of the TCE group, which had the highest myostatin expression compared to the control group, is likely attributed to high *MYOG* expression with the lowest variability of this trait among all the compared groups.

It should be noted that the expression of the studied genes in the experimental groups was characterized by high variability, predominately with regard to myogenin expression. The MRE group showed the lowest homogeneity on this indicator, which, probably, can be related to the lowest content of extract in compound feeds. In this regard, the extract was not ingested in equal amounts, which had different effects on the gene transcriptional activity of individual birds. In addition, different expressions of myogenin in chickens of the experimental groups could be due to genetic features of chickens. In particular, SNPs in the *MYOG* gene of broiler chickens associated with meat productivity [48,49] were identified, which may cause different levels of gene expression and different sensitivity of expression to nutritional factors. In general, relatively high variability in the expression of genes related to muscle growth and differentiation, including *MYOG* and *MSTN*, was described in other studies under the influence of both paratypical [50] and genetic factors [51]. The high variability of *FASN* expression in the CCE group is probably due to the fact that inulin, as a nutrient substrate for microbiota, influenced the metabolism and lipogenesis of poultry indirectly through complex mechanisms of interaction between the gastrointestinal microbiome and the macroorganism, while phytochemicals in the SJWE, MRE, and TCE groups were absorbed in the intestine, entered the bloodstream, and participated directly and topically in the processes of fat metabolism activation.

The comparison of *MYOG* expression levels and uric acid concentration in the experimental groups showed a pronounced inverse correlation, indicating a more intense utilization of protein metabolism metabolites with higher *MYOG* transcriptional activity.

The relationship between trypsin activity and the mass of the pancreas and intestine proved to be of interest. In the TCE group, the highest mass of these organs was observed alongside the lowest trypsin activity, while in the SJWE group, this relationship was reversed. It can be assumed that with the increase in pancreatic enzyme function, the digestion and absorption of nutrients in the feed occur more intensively, leading to a reduction in chyme presence in the intestinal tract, subsequently reducing its size.

The obtained results indicate the presence of growth-stimulating properties of the studied plant extracts, which were revealed both by the results of monitoring of live weight and meat productivity of broiler chickens, and by the results of molecular genetic studies of transcriptional activity of myo- and lipogenesis genes. Further studies will be aimed at identifying other biologically active properties of the extracts, which, in combination with the available data, will allow for the development of an optimal composition of phytocombination based on the described phytobiotics. However, according to the results of this study, taking into account the reliable increase in the live weight of chickens in the initial period of ontogenesis, the tendency to increase muscle weight, and the statistically significant increase in the expression of *MYOG* and *FASN* genes with no significant increase in the expression of *MSTN*, it can be recommend to use the extract of lewesia safflower (*R. carthamoides* L.) in the nutrition of broiler chickens in the amount of 170 g/t in Starter and Grower feeds and 210 g/t in Finisher feed.

## 5. Conclusions

Thus, growth-stimulating properties of bioactive compounds of plant origin were identified, manifested in the increase in live weight of broiler chickens and activation of genes associated with the growth and development of skeletal muscles (*MYOG*) and fatty acid synthesis (*FASN*), as well as intensification of metabolism. The increased expression of *MSTN* under the influence of biologically active components included in the extract of creeping thyme substantiates the feasibility of further studies to determine the optimal level of inclusion of this extract. The obtained data are nutrigenomic justification for the development of a new phytocombination for increasing meat productivity of broiler chickens based on the optimal ratio of extracts of common chicory (*Cichorium intubus* L.), St. John’s wort (*Hypericum perforatum* L.), safflower (*Rhaponticum carthamoides* L.), and creeping thyme (*Thymus serpyllum* L.).

## Figures and Tables

**Table 1:** Feeding scheme and research design.

Groups	Poultry Number (*n*)	Broiler Chicken Feeding Program
CON	36	Basic diet (BD) without phytochemicals
CCE	36	BD + CCE 450 g/t in Starter and Grower, 560 g/t Finisher
SJWE	36	BD + SJWE 350 g/t in Starter and Grower, 430 g/t Finisher
MRE	36	BD + MRE 170 g/t in Starter and Grower, 210 g/t Finisher
TCE	36	BD + TCE 200 g/t in Starter and Grower, 250 g/t Finisher

CON—control; CCE—common chicory extract; SJWE—St. John’s wort extract; MRE—maral root extract; and TCE—thyme creeping extract.

**Table 2:** Nutritional value of compound feeds for broiler chickens.

Nutrients	Age of Poultry (Days)		
	0–10 (Starter)	11–22 (Grower)	23–35 (Finisher)
	Nutritional Value (%)		
Metabolic energy (ME) (kcal/100 g)	305.00	299.00	303.00
Crude protein	23.00	21.50	18.50
Assimilable lysine	1.44	1.33	1.20
Assimilable methionine	0.67	0.67	0.63
Assimilable methionine + cystine	1.04	1.03	0.94
Assimilable threonine	0.97	0.91	0.81
Assimilable tryptophan	0.26	0.22	0.21
Crude fiber	3.69	4.75	5.00
Essential extract	3.73	5.38	5.50
Linoleic acid	1.74	2.63	2.81
Calcium	1.10	0.87	0.78
Total phosphorus	0.69	0.67	0.61
Assimilable phosphorus	0.58	0.42	0.51
Sodium	0.17	0.17	0.18
Chlorine	0.17	0.19	0.20
Vitamin B4 (mg/kg)	1568.00	1568.00	1540.00

**Table 3:** Nucleotide sequence of primers.

Gene	Primers	Author
*ACTB* (β-actin)	F: CTGTGCCCATCTATGAAGGCTA R: ATTTCTCTCTCGGCTGTGGTG	Laptev G.Yu. et al., 2023 [19]
*MYOG* (myogenin)	F: GGAGAAGCGGAGGCTGAAG R: GCAGAGTGCTGCGTTTCAGA	Laptev G.Yu. et al., 2022 [18]
*MSTN* (myostatin)	F: GCTTTTGATGAGACTGGACGAG R: AGCGGGTAGCGACAACATC	Dou T. et al., 2018 [29]
*FASN* (fatty acid synthase)	F: GCAGCTTCGGTGCCTGTGGTT R: GCTGCTTGGCCCACACCTCC	Kishawy A.T.Y. et al., 2022 [30]

**Table 4:** Dynamics of live weight of broiler chickens, g.

Age of Poultry	CON	Group				*p*-Value
		CCE	SJWE	MRE	TCE	
1 day (initial)	42.1 ± 0.50	42.1 ± 0.48	41.7 ± 0.49	41.9 ± 0.49	42.3 ± 0.56	0.956
7 days	188.0 ± 3.93 ^a^	200.6 ± 5.27 ^ab^	200.2 ± 4.14 ^ab^	214.1 ± 3.32 ^b^	200.8 ± 3.85 ^ab^	0.001
14 days	513.0 ± 10.87 ^a^	536.8 ± 12.41 ^ab^	540.1 ± 11.18 ^ab^	561.1 ± 7.88 ^b^	542.0 ± 8.48 ^ab^	0.031
21 days	1091.5 ± 22.64	1088.5 ± 22.15	1083.3 ± 20.76	1109.0 ± 18.51	1089.2 ± 16.51	0.910
28 days	1581.7 ± 35.92	1655.0 ± 36.10	1634.8 ± 38.36	1655.6 ± 40.45	1645.8 ± 30.79	0.612
35 days (final)	2061.9 ± 51.62	2204.6 ± 50.38	2167.3 ± 55.63	2216.1 ± 48.91	2146.8 ± 44.50	0.233

Values are expressed as means ± standard error. Means denoted within the same row with different superscripts are significant (*p* < 0.05).

**Table 5:** The weight of organs of broiler chickens (*Gallus gallus* L.) of the “Smena 9” cross when using phytobiotics in the diet, g.

Parameters	CON	Groups				*p*-Value
		CCE	SJWE	MRE	TCE	
Carcass components						
Total sample (*n* = 6)						
Skeletal muscles (g)	853.0 ± 15.33	925.3 ± 48.16	936.7 ± 25.51	965.2 ± 34.62	961.3 ± 33.44	0.149
Skin with fat * (g)	168.2 ± 7.22 ^a^	176.2 ± 5.45 ^ab^	205.4 ± 4.90 ^b^	199.0 ± 12.99 ^ab^	200.8 ± 6.13 ^ab^	0.008
Bones (g)	372.7 ± 37.15	412.0 ± 27.12	357.7 ± 22.47	360.3 ± 18.26	370.2 ± 43.23	0.739
♂ (*n* = 3)						
Skeletal muscles (g)	881.7 ± 10.14	1011.0 ± 57.86	982.3 ± 17.89	1023.8 ± 34.79	1030.8 ± 25.00	0.056
Skin with fat * (g)	177.6 ± 7.55	182.9 ± 5.47	205.2 ± 10.41	220.0 ± 17.95	201.3 ± 11.10	0.127
Bones (g)	431.9 ± 48.41	451.4 ± 24.43	399.3 ± 23.91	384.9 ± 12.23	442.8 ± 61.17	0.700
♀ (*n* = 3)						
Skeletal muscles (g)	824.2 ± 15.65	839.5 ± 29.91	891.03 ± 29.16	906.6 ± 36.69	891.8 ± 11.65	0.187
Skin with fat * (g)	158.9 ± 10.80 ^a^	169.4 ± 8.53 ^ac^	205.6 ± 3.40 ^bc^	178.0 ± 8.89 ^ac^	200.3 ± 8.03 ^bc^	0.011
Bones (g)	313.6 ± 32.54	372.6 ± 39.08	316.1 ± 14.91	335.8 ± 30.22	297.5 ± 18.02	0.440
Gastrointestinal tract						
Total sample (*n* = 6)						
Esophagus and crop (g)	14.5 ± 1.29	26.1 ± 7.29	13.5 ± 1.98	15.7 ± 1.85	14.6 ± 1.51	0.112
Glandular stomach (g)	11.7 ± 1.74	11.2 ± 1.70	9.5 ± 1.52	10.4 ± 1.55	8.9 ± 0.82	0.662
Muscular stomach (g)	19.6 ± 2.23	21.9 ± 1.58	16.2 ± 0.61	19.2 ± 2.36	15.1 ± 1.32	0.069
Gizzard lining (g)	3.3 ± 0.49 ^a^	4.8 ± 0.33 ^b^	3.7 ± 0.36 ^ab^	3.5 ± 0.31 ^ab^	2.7 ± 0.30 ^a^	0.007
Liver (g)	39.3 ± 2.08	41.6 ± 1.92	43.7 ± 2.75	41.1 ± 2.15	42.0 ± 1.54	0.686
Gallbladder (g)	1.5 ± 0.15	1.3 ± 0.18	2.2 ± 0.50	2.0 ± 0.23	2.0 ± 0.15	0.124
Pancreas (g)	4.0 ± 0.32	4.1 ± 0.24	3.9 ± 0.22	4.1 ± 0.12	4.7 ± 0.33	0.277
Intestines (g)	72.4 ± 6.64 ^ab^	78.7 ± 2.57 ^ab^	58.4 ± 3.51 ^a^	66.4 ± 4.23 ^ab^	82.9 ± 9.40 ^b^	0.048
♂ (*n* = 3)						
Esophagus and crop (g)	15.7 ± 2.63	37.4 ± 11.48	17.6 ± 1.72	14.9 ± 2.36	16.7 ± 2.40	0.074
Glandular stomach (g)	15.5 ± 1.09	13.6 ± 2.89	11.3 ± 2.77	13.1 ± 2.19	9.3 ± 1.62	0.404
Muscular stomach (g)	23.7 ± 1.68	23.7 ± 2.75	16.4 ± 1.30	17.1 ± 2.13	16.7 ± 2.39	0.058
Gizzard lining (g)	4.1 ± 0.65	4.9 ± 0.54	4.3 ± 0.48	3.4 ± 0.66	2.7 ± 0.37	0.117
Liver (g)	40.8 ± 3.50	44.0 ± 2.67	48.7 ± 1.90	45.2 ± 2.43	43.9 ± 2.38	0.384
Gallbladder (g)	1.3 ± 0.18	1.4 ± 0.20	2.7 ± 0.93	2.2 ± 0.37	2.1 ± 0.29	0.267
Pancreas (g)	4.2 ± 0.54	4.3 ± 0.37	4.0 ± 0.25	4.0 ± 0.23	4.2 ± 0.33	0.971
Intestines (g)	81.1 ± 11.40	79.3 ± 5.03	65.0 ± 3.80	75.4 ± 3.04	78.2 ± 0.94	0.391
♀ (*n* = 3)						
Esophagus and crop (g)	13.4 ± 0.32	14.8 ± 2.32	9.5 ± 0.63	16.5 ± 3.32	12.6 ± 1.15	0.195
Glandular stomach (g)	8.0 ± 0.17	8.7 ± 0.20	7.6 ± 0.71	7.7 ± 0.25	8.4 ± 0.75	0.485
Muscular stomach (g)	15.5 ± 2.28	20.1 ± 1.33	15.9 ± 0.36	21.2 ± 4.39	13.6 ± 0.78	0.176
Gizzard lining (g)	2.4 ± 0.23 ^a^	4.7 ± 0.52 ^b^	3.0 ± 0.09 ^ab^	3.5 ± 0.42 ^ab^	2.7 ± 0.56 ^a^	0.011
Liver (g)	37.7 ± 2.63	39.2 ± 2.33	38.8 ± 3.11	37.1 ± 0.69	40.0 ± 1.56	0.885
Gallbladder (g)	1.7 ± 0.19	1.2 ± 0.34	1.7 ± 0.34	1.9 ± 0.32	1.9 ± 0.17	0.444
Pancreas (g)	3.8 ± 0.45	4.0 ± 0.36	3.8 ± 0.39	4.2 ± 0.09	5.2 ± 0.43	0.115
Intestines (g)	63.7 ± 3.88	78.1 ± 2.69	51.9 ± 2.08	57.4 ± 0.28	87.6 ± 20.48	0.114

Values are expressed as means ± standard error. Means denoted within the same row with different superscripts are significant (*p* < 0.05). * Subcutaneous and abdominal fat. ♂—roosters, ♀—hens.

**Table 6:** Blood biochemical parameters of broiler chickens (*Gallus gallus* L.) of the “Smena 9” cross when using phytochemicals in the diet.

Parameters	CON	Groups				*p*-Value
		CCE	SJWE	MRE	TCE	
Total protein, g/L	35.0 ± 0.21	25.6 ± 8.50	34.6 ± 0.79	34.2 ± 0.52	36.2 ± 0.92	0.374
Uric acid, mmol/L	359.9 ± 15.06 ^a^	140.4 ± 52.98 ^b^	74.3 ± 8.04 ^b^	66.5 ± 14.84 ^b^	102.7 ± 3.84 ^b^	0.000
Triglycerides, mmol/L	0.40 ± 0.043	0.34 ± 0.108	0.36 ± 0.012	0.28 ± 0.076	0.39 ± 0.033	0.704
Calcium, mmol/L	3.79 ± 0.132	3.47 ± 0.169	3.69 ± 0.097	3.39 ± 0.114	3.86 ± 0.092	0.056
Phosphorus, mmol/L	2.47 ± 0.063 ^a^	2.33 ± 0.107 ^ab^	2.14 ± 0.023 ^b^	2.34 ± 0.032 ^ab^	2.29 ± 0.034 ^ab^	0.010
Serum blood enzymatic activity						
Trypsin, U/L	63.8 ± 5.73	77.4 ± 5.36	86.4 ± 9.48	77.6 ± 3.37	62.7 ± 6.52	0.069
AST, U/L	238.1 ± 14.27	254.5 ± 9.13	202.3 ± 29.07	225.7 ± 12.90	229.4 ± 19.22	0.384
ALT, U/L	16.2 ± 1.25	13.2 ± 0.73	15.4 ± 1.87	16.8 ± 1.78	18.0 ± 2.17	0.340
Lipase, U/L	68.5 ± 0.57	73.4 ± 1.68	73.3 ± 1.38	73.1 ± 3.28	68.2 ± 1.25	0.117
Amylase, U/L	413.3 ± 20.95	345.0 ± 111.62	516.5 ± 22.50	617.5 ± 118.50	415.0 ± 19.36	0.222
Alkaline phosphatase, U/L	965.5 ± 19.04	451.8 ± 247.43	455.3 ± 246.62	260.7 ± 162.03	467.8 ± 86.35	0.133

Values are expressed as means ± standard error. AST—aspartate aminotransferase. ALT—alanine aminotransferase. Means denoted within the same row with different superscripts are significant (*p* < 0.05).

## Data Availability

The raw data supporting the conclusions of this article will be made available by the authors without undue reservations. The data presented in this study are available upon request from the corresponding author.
